# *bSiteFinder*, an improved protein-binding sites prediction server based on structural alignment: more accurate and less time-consuming

**DOI:** 10.1186/s13321-016-0149-z

**Published:** 2016-07-11

**Authors:** Jun Gao, Qingchen Zhang, Min Liu, Lixin Zhu, Dingfeng Wu, Zhiwei Cao, Ruixin Zhu

**Affiliations:** Department of Bioinformatics, Tongji University, Shanghai, 200092 People’s Republic of China; School of Information Engineering, Shanghai Maritime University, Shanghai, 201306 People’s Republic of China; Digestive Diseases and Nutrition Center, Department of Pediatrics, The State University of New York at Buffalo, Buffalo, NY 14260 USA; Genomics, Environment, and Microbiome Community of Excellence, The State University of New York at Buffalo, Buffalo, NY 14203 USA; Institute of Digestive Diseases, Longhua Hospital, Shanghai University of Traditional Chinese Medicine, Shanghai, 200032 People’s Republic of China

**Keywords:** Protein-binding sites prediction, Structural alignment, Multiple-Templates Clustering, Index, Web server

## Abstract

**Motivation:**

Protein-binding sites prediction lays a foundation for functional annotation of protein and structure-based drug design. As the number of available protein structures increases, structural alignment based algorithm becomes the dominant approach for protein-binding sites prediction. However, the present algorithms underutilize the ever increasing numbers of three-dimensional protein–ligand complex structures (bound protein), and it could be improved on the process of alignment, selection of templates and clustering of template. Herein, we built so far the largest database of bound templates with stringent quality control. And on this basis, *bSiteFinder* as a protein-binding sites prediction server was developed.

**Results:**

By introducing Homology Indexing, Chain Length Indexing, Stability of Complex and Optimized Multiple-Templates Clustering into our algorithm, the efficiency of our server has been significantly improved. Further, the accuracy was approximately 2–10 % higher than that of other algorithms for the test with either bound dataset or unbound dataset. For 210 bound dataset,* bSiteFinder* achieved high accuracies up to 94.8 % (MCC 0.95). For another 48 bound/unbound dataset,* bSiteFinder* achieved high accuracies up to 93.8 % for bound proteins (MCC 0.95) and 85.4 % for unbound proteins (MCC 0.72). Our *bSiteFinder* server is freely available at http://binfo.shmtu.edu.cn/bsitefinder/, and the source code is provided at the methods page.

**Conclusion:**

An online *bSiteFinder* server is freely available at http://binfo.shmtu.edu.cn/bsitefinder/. Our work lays a foundation for functional annotation of protein and structure-based drug design. With ever increasing numbers of three-dimensional protein–ligand complex structures, our server should be more accurate and less time-consuming.

**Graphical Abstract:**
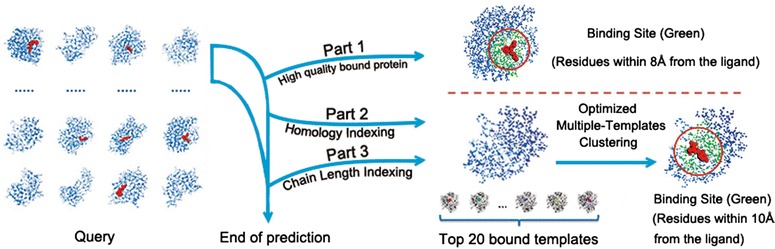
*bSiteFinder* (http://binfo.shmtu.edu.cn/bsitefinder/) as a protein-binding sites prediction server was developed based on the largest database of bound templates so far with stringent quality control. By introducing Homology Indexing, Chain Length Indexing, Stability of Complex and Optimized Multiple-Templates Clustering into our algorithm, the efficiency of our server have been significantly improved. What’s more, the accuracy was approximately 2–10 % higher than that of other algorithms for the test with either bound dataset or unbound dataset

## Background

Most biological processes involve the interaction of ligands with proteins. Functional characterization of ligand-binding sites of proteins is a key issue in understanding those biological processes [1–4]. In addition, identifying the location of protein-binding sites is a vital first step in structure-based drug design [5–8]. However, functional characterization of proteins through experimental method is a labor intensive and time-consuming process. A computational tool to predict the functional binding sites in a protein is therefore of practical importance.

To date, a variety of computational methods have been developed for protein-binding sites prediction, which can be divided into four categories: geometry based methods [9–14], energy based methods [15, 16], alignment based methods [17–20] and other miscellaneous methods [21–23]. Alignment based methods can be further divided into sequence alignment based and structural alignment based methods. Recently, increasing structural genomics projects have led to the exponential growth of the number of available protein structures. As a consequence, structural alignment based methods exceeded other methods due to its more efficient and more accurate performance.

In 1996, Lichtarge et al. [17] developed the first structural alignment based algorithm for protein-binding sites prediction, entitled evolutionary trace method (ET method). It is based on the extraction of functionally important residues from sequence conservation patterns in homologous proteins, and on their mapping onto the protein surface to generate clusters identifying functional interfaces. In 2007, Brylinski and Skolnick developed a popular structural alignment method called FINDSITE [18]. For a given target sequence, FINDSITE identifies ligand-bound template structures from a set of distantly homologous proteins recognized by the PROSPECTOR_3 threading approach and superposes them onto the target’s structure using the TM-align structural alignment algorithm. Binding pockets are identified by the spatial clustering of the center of mass of template-bound ligands that are subsequently ranked by the number of binding ligands. In 2009, Oh et al. [24] developed LEE, a two-stage template-based ligand binding site prediction method, where templates are used first for protein 3D modeling and then for binding site prediction by structural clustering of ligand-containing templates to the predicted 3D model. Later in 2010, Wass et al. [25] described a new method called 3DligandSite. Structures similar to the query are identified by using MAMMOTH [26] against a library of protein structures with bound ligands. The structural based alignment of the similar structures and the query superposes ligands onto the query structures. After filtering, the top 25 ligands are retained for analysis and further clustering. In 2012, another comparative approach called COFACTOR was proposed by Zhang group [19]. COFACTOR recognizes functional sites of protein–ligand interactions using low-resolution protein structural models, based on a global-to-local sequence and structural comparison algorithm. The major advantage of COFACTOR over the existing methods is the optimal combination of global and local structural comparisons for identifying protein-binding sites. But, the global comparison can be distracted by structural variations in the regions far away from the binding pockets; meanwhile the local comparison has a high false positive rate since the number of residues involved is too small. Later in 2013, Zhang group published another structural alignment based algorithm, TM-SITE [20]. Different from COFACTOR, TM-SITE compares the structures of a subsequence from the first binding residue to the last binding residue (called SSFL) on the query and template proteins, which solve the problems of global-to-local structural comparison algorithm. These methods provide us valuable choices to predict the binding sites. However, their performance needs to be improved for lack of accuracy or time-efficiency or both since the structural information of protein–ligand complexes (bound protein) are underutilized.

Herein, we built so far the largest database of bound templates with stringent quality control. And on this basis, *Stability of Complex* as a new criterion and Optimized Multiple-Templates Clustering algorithm are introduced to improve the accuracy. Meanwhile, Homology Indexing and Chain Length Indexing are used to accelerate the efficiency of the structural alignment. Finally, we presented a user friendly protein-binding sites prediction web server (*bSiteFinder*), at http://binfo.shmtu.edu.cn/bsitefinder/.

## Methods

### Definitions of operations

#### Rules of five

The protein data in PDB database are filtered through the rules below:

1. The macromolecule type is protein, no DNA and RNA.
2. Experiment method is set to X-ray.
3. X-ray resolution is between 0 and 3.0.
4. Has free ligands = yes.
5. Sequence length is over 20.

#### Number of ligand atoms

In the process of building databases, which database a protein finally falls into depends on whether it contains ligands and whether these ligands have enough atoms. For this reason, ligands identification, which is judged by the rules mentioned below, plays a key role. Every HETATM residue is recognized through HET records from the header of PDB files. Notably, some of the residues are modified on normal chains, which are not counted as true ligands because of their present in the MODRES records. Hence, the selected ligands only come from HET records excluding MODRES ones. Water molecule is included in HETATM but not regarded as a ligand. Analyzing the data, we define that a ligand should possess 6 or more atoms as a basic rule to identify a ligand.

#### Stability of Complex

The binding site check criterion is using as the standard of judging the bound structure’s stability. Only if any one of atoms of the ligand has a distance within 4 Å from the geometry center of the calculated binding site, the structure of complex is considered to be stable.

#### Homology Indexing

Homology Indexing is implemented by using SCOPe, version 2.03 [27]. First, a four-digit classification number is searched based on PDB ID and CHAIN ID of the query chain. After that, all the protein chains with the same classification number are obtained and used to constitute the template database for subsequent structural alignment.

#### Chain Length Indexing

Only the chains, which have length difference with query chain less than 30 %, are used as candidates for subsequent structural alignment.

#### Structural alignment

The structural alignment between query and templates in *bSiteFinder* is implemented by using Combinatorial Extension (CE) algorithm, which is provided by Biojava [28]. Different from traditional dynamic programming algorithm and Monte Carlo algorithm, CE algorithm defines continuous residues in the sequence as aligned fragment pairs (AFPs), which is used in local alignment between query and template. Finally, the optimized alignment results are obtained by expanding or abandoning the local AFPs.

#### Optimized Multiple-Templates Clustering

**Fig. 1:**
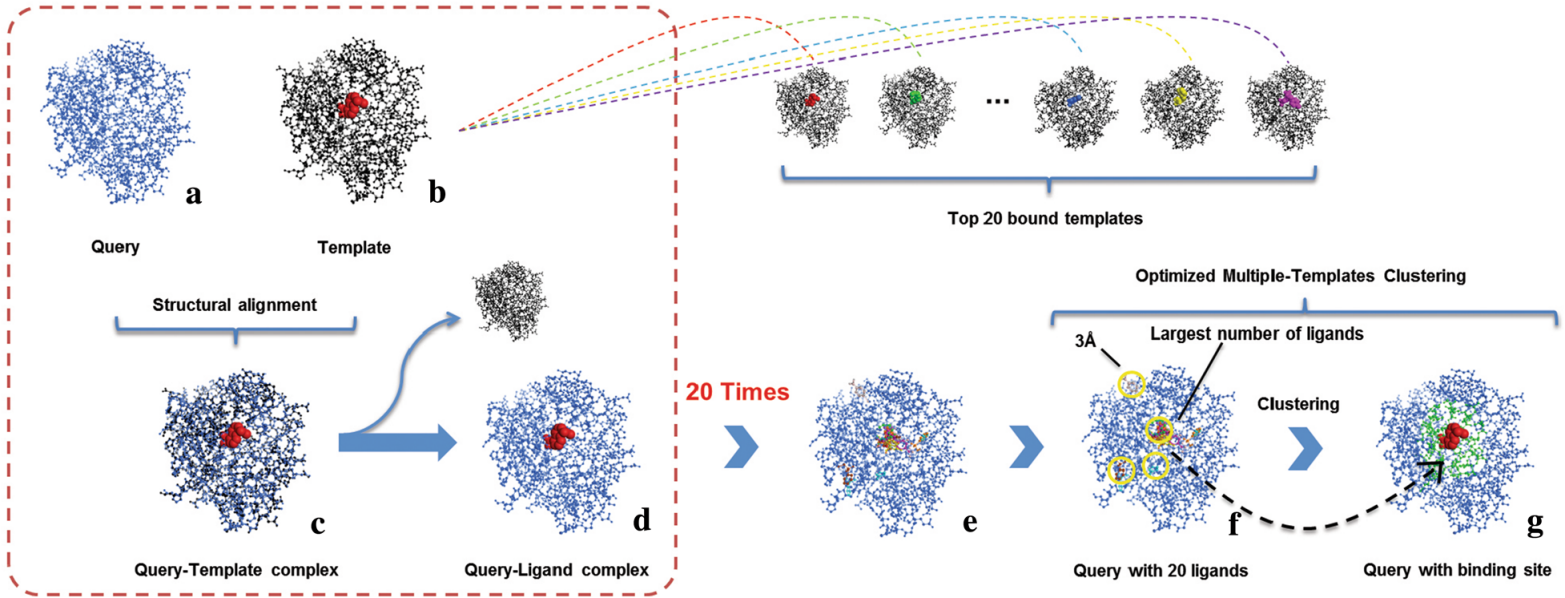
Workflow of Optimized Multiple-Templates Clustering. Template (*b*) is mapped to query (*a*) by structural alignment to form query-template complex (*c*). Then, the template chain will be removed, and the ligand will be retained (*d*). After 20 times of structural alignments, the ligands in templates will be mapped to the query (*e*). The number of ligand geometric centers, which have a distance less than 3 Å from the certain ligand geometric center, is counted for each ligand (*f*). The ligand with the largest number is defined as the center of the Top1 binding site (*g*)

After structural alignment, template will be mapped to query. Then, the templates which meet the requirement of Stability of Complex are ranked according to the similarity with query chain, and ligands of the top 20 templates at most will be picked out. After 20 times of structural alignments, all the ligands in templates will be mapped to the query. Further, these ligands are clustered into different clusters. The number of ligand geometric centers, which have a distance less than 3 Å from the certain ligand geometric center, is counted for each ligand. After that, the ligand with the largest number is defined as the center of the Top1 binding site (Fig. 1). Then, this ligand and all the other ligands within 3 Å are removed for searching the centers of the Top2 and Top3 binding site in the same way.

#### Detection of binding sites

On the condition that protein chains have ligands, we define all residues within the distance of 8 Å from ligands as the components of the binding site. On the condition that binding site is detected by doing structural alignment with templates, all residues within the distance of 10 Å from mapped ligands are defined as the components of the binding site. It should be noted that if the bound proteins’ stabilities did not pass the evaluation of *Stability of Complex*, the bound proteins would be treated as unbound proteins with original ligands removed.

### Test and evaluation methods

For comparing with other binding site prediction algorithms, two widespread adopted datasets from LIGSITEcsc [29] were used for testing our algorithm with the same criteria of evaluating the accuracy of binding site prediction. The first test set contained 210 proteins with ligands (bound dataset). At the suggestion of RCSB, protein 1B6N was replaced by 1Z1H. The second test set contained 48 proteins with/without ligands (bound/unbound dataset).

Here, the accuracy and Matthews Correlation Coefficient (MCC) [30] were both used to evaluate our algorithm.

#### Accuracy

A widely accepted verification method [13] was used. For bound protein, if the protein–ligand’s stability has passed the evaluation of *Stability of Complex*, the accuracy is 100 %. If the protein–ligand’s stability did not pass the evaluation of *Stability of Complex*, the original ligands of bound protein will be removed and in this situation, the bound protein will be regarded as unbound protein and may have a lower accuracy.

For unbound proteins, if the geometric center of a binding site has a distance within 4 Å from any one of the atoms of the predicted ligands, this binding site is regarded as a correctly predicted binding site. Otherwise, this binding site is regarded as an incorrectly predicted binding site.

#### MCC

Another evaluation index, MCC, was also used to evaluate the accuracy of binding site prediction. For each protein chain, all the residues were divided into four categories: TP: correctly predicted binding site residues; TN: correctly predicted nonbinding site residues; FP: incorrectly predicted as binding site residues; and FN: incorrectly predicted as nonbinding site residues. MCC scores are defined as:

1 $$MCC = \frac{TP \times TN - FP \times FN}{{\sqrt {(TP + FP) \times (TP + FN) \times (TN + FP) \times (TN + FN)} }}$$

For bound proteins that passed the evaluation of *Stability of Complex*, the MCC is 1. Otherwise, the bound proteins was regarded as unbound proteins and MCC would be lower than 1.

For unbound proteins, the structural alignment between query and template is implemented to map the ligands in bound proteins to the unbound proteins. Then, the mapped **pseudo** ligands were used to detect the binding site as describe in “*Detection of Binding Sites*”. To evaluate our methods, we divided the residues of query chains into residues of predicted binding site (Res-BS-Pre) and residues of predicted non-binding site (Res-NBS-Pre). At the same time, we also define residues of experimental binding site as Res-BS-Exp and residues of experimental non-binding site as Res-NBS-Exp according to the original ligands of query chains. Therefore, in formula (1), TP is the intersection of Res-BS-Pre and Res-BS-Exp, and TN is the intersection of Res-NBS-Pre and Res-NBS-Exp, and FP is the intersection of Res-BS-Pre and Res-NBS-Exp, and FN is the intersection of Res-NBS-Pre and Res-BS-Exp.

## Experimental

### Create template database

**Fig. 2:**
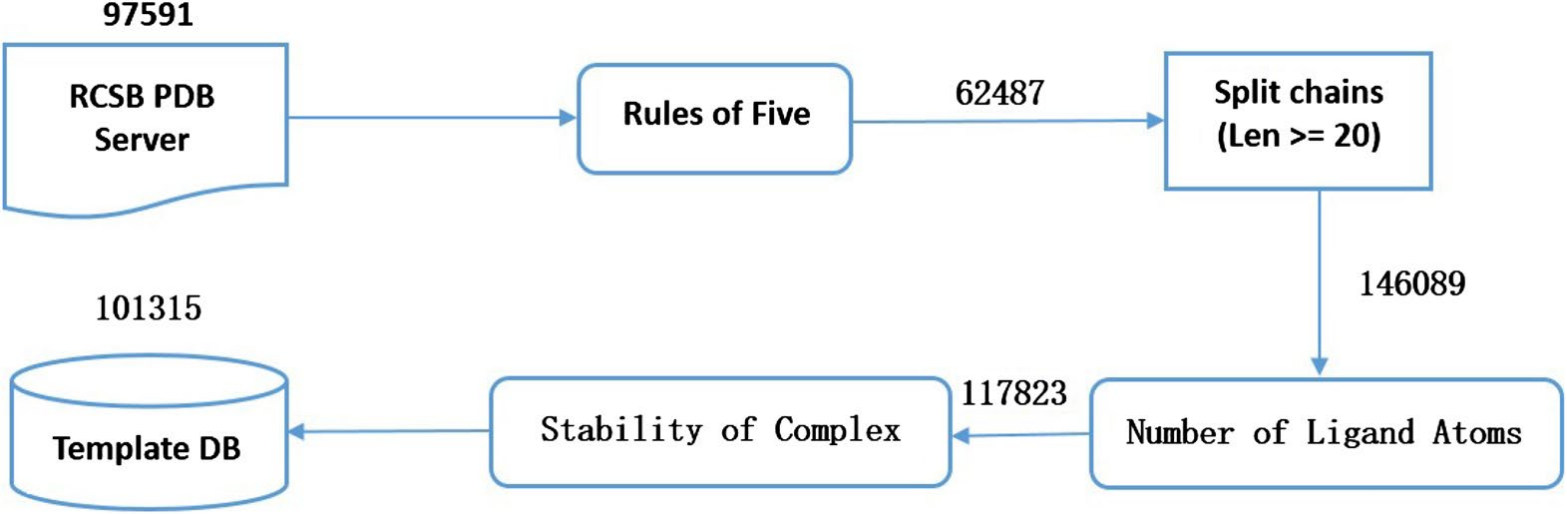
Workflow of creating template database

Our algorithm will maximize the information of bound proteins. Herein, we built so far the largest database of bound templates from PDB database with stringent quality control. Figure 2 shows the workflow of creating template database, which include four steps as follow: (1) 97,591 complex structures in PDB database (February 11, 2014) were filtered according to *Rules of Five*, and 62,487 complex structures were obtained. (2) Proteins were divided into chains, and then the chains which are less than 20 residues in length were removed. After that, 146,089 chains were obtained. (3) *Number of Ligand Atoms* was employed to ensure that there is at least one ligand in the complex structures of each chain, and 117,823 chains were obtained. (4) *Stability of Complex* was employed to ensure that it forms a stable bound structure of each chain with its ligand. Finally, 101,315 chains were obtained for building the database of bound templates.

### Workflow of binding sites detection

**Fig. 3:**
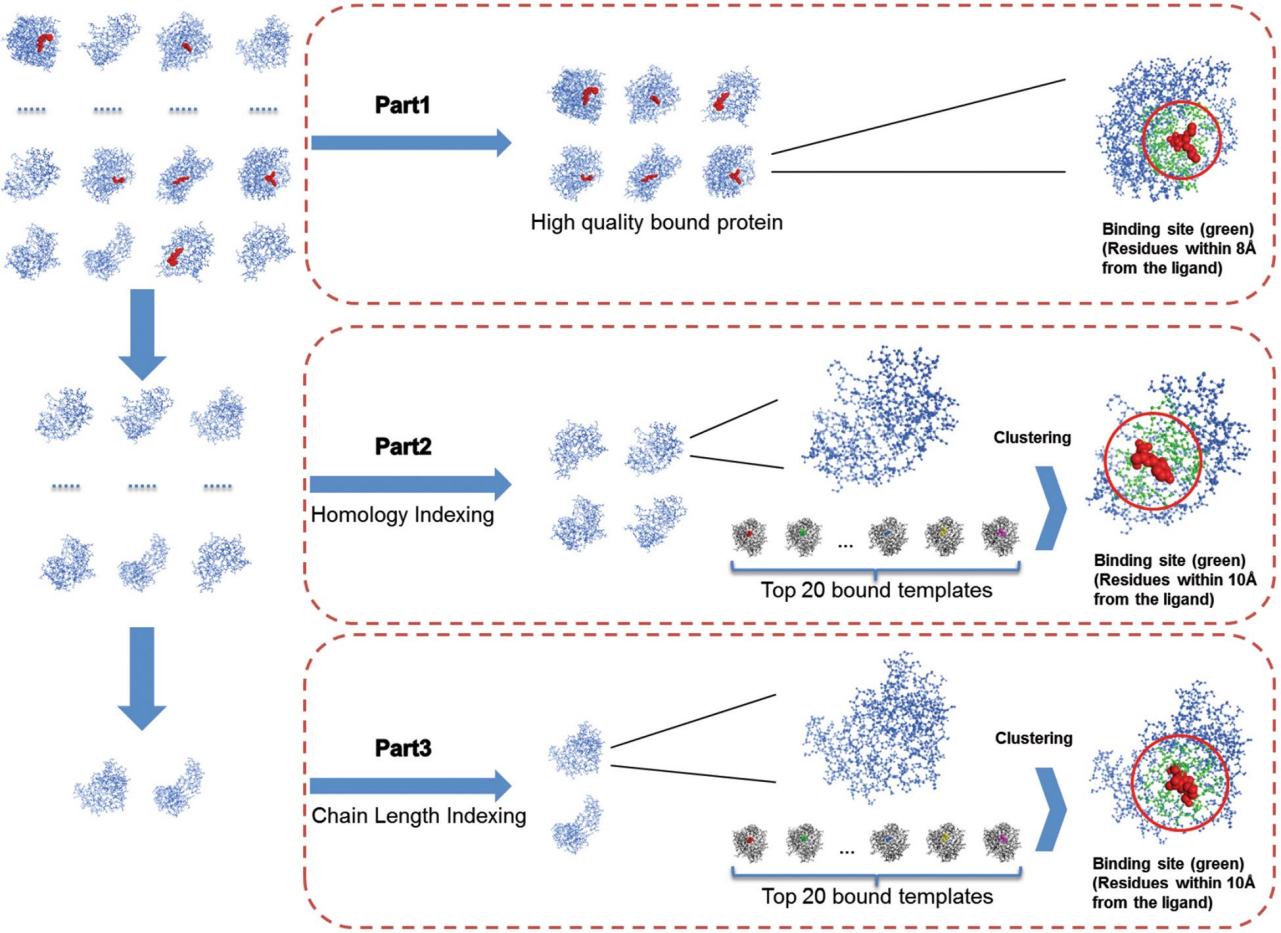
Workflow of binding sites detection. Each protein chain submitted would be processed successively by following steps: *1* Binding sites prediction of high quality bound protein (Part 1), or enter the following process. *2* Binding sites prediction of unbound protein with bound templates of same Homology Indexing (Part 2), or enter the following process. *3* Binding sites prediction of unbound protein with bound templates of Chain Length Indexing (Part 3). Any protein chains submitted into our system could receive the results of binding sites via efficient computation

When a query protein is submitted by user for binding site prediction, it will be firstly divided into chains. After that, the prediction will be done for each chain. Figure 3 shows the workflow of binding sites detection. Each protein chain will be processed by following steps:

1. Binding sites prediction of high quality bound protein (Part 1)

*Detection of Binding Sites* is employed for binding site detection, when the protein chains meet the requirement of *Number of Ligand Atoms* and *Stability of Complex*. Otherwise, enter the following process.

2. Binding sites prediction of unbound protein with bound templates of same Homology Indexing (Part 2)

If the query chain has a four-digit classification number in SCOPe and has bound template with the same Homology Indexing in template database, the binding site of this query chain will be detected as the following procedure. First, structural alignments between query chain and templates will be done, and the top 20 bound templates which are the most similar to the query will be selected subsequently. The locations of ligands are detected by mapping the ligands in templates to the query, and then the optimization of binding sites was following by using the new developed *Optimized Multiple*-*Templates Clustering* method. Finally, *Detection of Binding Sites* will be employed for binding site detection. Otherwise, enter the following process.

3. Binding sites prediction of unbound protein with bound templates of Chain Length Indexing (Part 3)

If the query chain has no satisfactory homologous bound template, the binding site of this query chain will be detected as the following procedure. Chain Length Indexing will be employed to search the bound templates, which have difference with query chain less than 30 % in length, in template database. Then enter the process as the description above (Part 2 of “Workflow of binding sites detection”) with top 20 most similar bound templates. Any protein chains submitted into our system could receive the results of binding sites via efficient computation.

## Results and discussion

### Performance of our algorithm and its comparison with others

Two widely adopted datasets including 210 bound and 48 bound/unbound dataset [29] were used for testing our algorithm, and the results are shown in Tables 1 and 2. The accuracy of our algorithm is approximately 2–10 % higher than that of other algorithms for the test with either bound or unbound datasets. In addition, with size of the dataset increased, our algorithm exhibited even more advantage over others regarding accuracy (The accuracy differences between our algorithm and the second highest algorithm in the Top1 increase from 2.4 % with 48 unbound dataset to 11.8 % with 210 unbound dataset).

**Table 1:** Comparison of the top1 and top3 success rates for various methods using 210 bound structures

Method	Top1^a^ (%)	Top3^a^ (%)
*bSiteFinder*	*94.8*	*95.7*
LISE^b^	83	94
MPK2^b^	81	95
MPK1^b^	75	93
Q-SiteFinder^b^	70	90
LIGSITE^CSCb^	75	–
LIGSITE^CSb^	70	86
PASS^b^	51	80
SURFNET^b^	42	57

^a^The MCC scores of the Top1 and Top3 are 0.95 and 0.97 respectively with 210 bound structures

^b^The success rates of these methods were taken from Xie and Hwang [32]

**Table 2:** Comparison of the top1 and top3 success rates for various methods using 48 bound/unbound structures

Method	Bound^a^		Unbound^b^	
	Top1 (%)	Top3 (%)	Top1 (%)	Top3 (%)
*bSiteFinder*	*93.8*	*98.7*	*85.4*	*95.8*
LISE^c^	92	96	81	92
MPK2^c^	85	96	80	94
VICE^c^	85	94	83	90
MPK1^c^	83	96	75	90
DoGSite^c^	83	92	71	92
Fpocket^c^	83	92	69	94
LIGSITE^CSc^	81	92	71	85
LIGSITE^CSCc^	79	–	71	–
MSPocket^c^	77	94	75	88
POCASA^c^	77	90	75	92
Q-SiteFinder^c^	75	90	52	75
PocketPicker^c^	72	85	69	85
CAST^c^	67	83	58	75
PASS^c^	63	81	60	71
SURFNET^c^	54	78	52	75

^a^The MCC scores of the Top1 and Top3 are 0.95 and 0.97 respectively with 48 bound structures

^b^The MCC scores of the Top1 and Top3 are 0.72 and 0.75 respectively with 48 unbound structures

^c^The success rates of these methods were taken from Xie and Hwang [32]

**Fig. 4:**
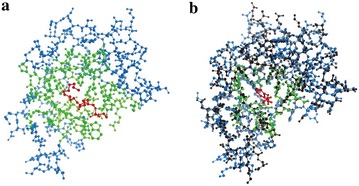
**a** Binding site of bound chain (PDB ID: 5p2p, CHAIN ID: A). The binding site is composed of residues (*green*) within 8 Å from the ligand (*red*). **b** Binding site of unbound chain (PDB ID: 3p2p, CHAIN ID: A, *blue*). The detection of binding site is based on the bound template (PDB ID: 1oxr, CHAIN ID: A, *black*) by mapping the ligand of template into unbound chain. And the binding site is composed of residues (*green*) within 10 Å from the ligand (*red*), which is different from 8 Å for bound chain prediction

For bound chain (such as PDB ID: 5p2p, CHAIN ID: A), the binding site is composed of residues within 8 Å from the ligand (Fig. 4a). For unbound chain (such as PDB ID: 3p2p, CHAIN ID: A), unlike bound chain, the binding site is detected with the aid of templates (PDB ID: 1oxr, CHAIN ID: A). First, the ligand in template is mapped to unbound chain. Then the binding site is composed of residues within 10 Å from the ligand (Fig. 4b). See Method part for details.

### Indexed alignment

Since there are still lots of protein chains have no satisfactory bound structures, bound templates is borrowed for detecting the binding sites in this situation. Our templates database contains 101,315 bound templates. It would consume a large amount of computation for predicting the binding site if structural alignments go through all the chains in the database. Thus, to improve the efficiency of our algorithm, Homology Indexing is introduced and then the time-consuming structural alignment will be limited only among homologous proteins. After building Homology Indexing for all 101,315 chains in template database by using SCOPe [27], 4254 protein classes are obtained. It means that only about 24 (101,315/4254) bound templates are needed to do the time-consuming structural alignment with the query per prediction. This would significantly reduce the computation time.

Table 3 shows the alignment frequency between templates and the query from the 48 unbound dataset after Homology Indexing is used. Without Homology Indexing, 48 unbound dataset should be aligned with each of chains in template database, which means that there are 48 × 101,315 time-consuming structural alignments needed to be done. But, with the Homology Indexing introduced, it can be reduced to 25,127 structural alignments, which only account for only 0.5 % of that without Homology Indexing. It’s worth noting that alignment frequencies, in Table 3, reach hundreds or even thousands in practical, which may be due to the uneven distribution of different protein families in template database at present.

**Table 3:** Frequency of structural alignment with 48 unbound chains using Homology Indexing

PDB ID CHAIN ID	Alignment frequency	PDB ID CHAIN ID	Alignment frequency	PDB ID CHAIN ID	Alignment frequency	PDB ID CHAIN ID	Alignment frequency
3tmsA	535	1ifbA	171	1cgeA	273	1bbsA	747
8adhA	424	3ptnA	1181	1hsiB	1203	1stnA	144
1hxfH	1326	1ypiA	170	1a4jB	489	1ptsA	268
2fbpA	269	5dfrA	463	1imeA	173	2ctbA	106
1gcgA	169	3phvA	1153	1nnaA	416	2cbaA	522
1helA	203	2ctvA	625	1ahcA	188	1krnA	6
1npcA	154	5cpaA	106	2tgaA	1176	2silA	377
1esaA	1246	1a6uH	397	4ca2A	523	1l3fE	156
1brqA	344	1qifA	567	1pdyA	56	1chgA	1160
8ratA	173	3appA	753	1phcA	873	6insE	124
1swbA	269	1djbA	620	1psnA	744	3p2pA	209
1ulaA	724	1byaA	78	3lckA	3131	7ratA	167

Although the efficiency of binding-sites prediction for unbound chains has been significantly increased benefiting from Homology Indexing, there are still some chains of no satisfactory homologous template structures, such as PDB ID: 4h12, CHAIN ID: A. For this kind of protein chains, we further introduce Chain Length Indexing to reduce the number of time-consuming structural alignments. Table 4 shows the alignment frequency between templates and the query from 20 dataset of no appropriate homologous templates after Chain Length Indexing is used. Without Chain Length Indexing, the 20 dataset of no homologous template chains should be aligned with each of chains in template database, which mean that there are 20 × 101,315 time-consuming structural alignments needed to be done. But, with the Chain Length Indexing introduced, it can be reduced to 663,739 structural alignments, which only account for 32.8 % of the number without Chain Length Indexing (Table 4).

**Table 4:** Frequency of structural alignment in 20 no homologous template chains with Chain Length Indexing involved

Query chain	Query length	Alignment frequency	Percentage of sequences passed Chain Length Indexing (%)	Query chain	Query length	Alignment frequency	Percentage of sequences passed Chain Length Indexing (%)
4ggbA	348	39,458	38.9	1wakA	353	39,014	38.5
2yzvA	286	39,291	38.8	4ff5A	227	31,362	31.0
4iezA	186	22,882	22.6	4fk9A	314	40,446	39.9
3ii7A	288	39,614	39.1	1ujcA	156	17,109	16.9
3a3jA	344	39,782	39.3	3ianA	319	40,000	39.5
3chlA	315	40,325	39.8	3rloA	196	25,455	25.1
2cf5A	352	39,192	38.7	3mfcA	187	23,369	23.1
2iq1A	257	36,141	35.7	3dgtA	278	38,641	38.1
1wy0A	327	40,200	39.7	2dh6A	331	40,195	39.7
2y7bA	134	14,998	14.8	1w4sA	146	16,265	16.1

It would be argued that the best template will be excluded by the use of Chain Length Indexing. However, the result indicates that, with or without Chain Length Indexing, there are no significant differences in the length between templates (Table 5).

**Table 5:** Top1 template for 20 no homologous template chains and their length obtained without Chain Length Indexing

Query chain	Query length	Template chain	Template length	Template chain (length constrained)	Template length
3mq1F	100	3mq1A	101	3mq1A	101
4kh0B	150	4kgvB	145	4kgvB	145
4fzbO	200	4fzbK	201	4fzbK	201
3ujoC	250	3ujoD	250	3ujoD	250
3zq6A	300	3zq6C	284	3zq6C	284
3mk6B	351	4ehtB	260	4ehtB	260
2q14B	400	2q14H	398	2q14H	398
2yg4B	450	2yg3A	449	2yg3A	449
4k3tA	498	4k3tB	498	4k3tB	498
4bthB	546	2wybB	546	2wybB	546
4mfdC	595	4jx5A	596	4jx5A	596
3szgA	650	3sytC	652	3sytC	652
3alaF	700	3alaE	701	3alaE	701
3w3lA	751	3w3lB	751	3w3lB	751
3lq4A	801	1rp7A	801	1rp7.A	801
3zhuD	852	2yidD	852	2yidD	852
2wyhB	891	2f7oA	1014	2f7oA	1014
2okxB	954	2okxA	954	2okxA	954
2xt6B	989	2xt6A	1055	2xt6A	1055
4dx5A	1044	2j8sA	1044	2j8sA	1044

### Stability of Complex

**Fig. 5:**
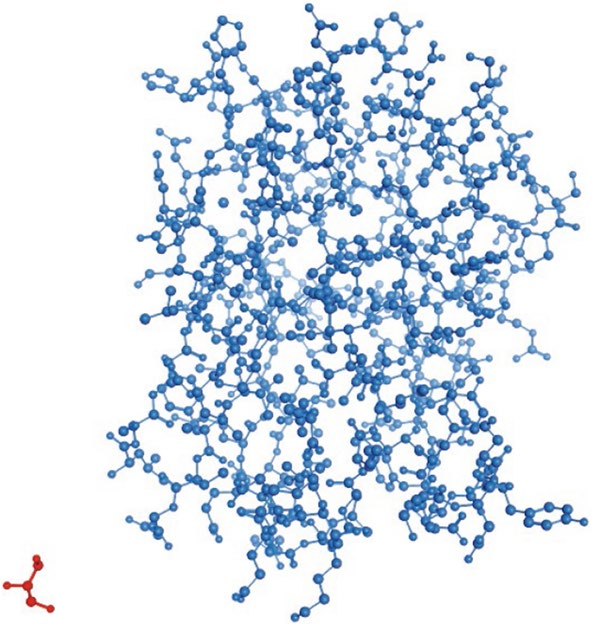
Unstable bound structure of ligand (GOL, *red*) and protein chain (PDB ID: 2j22, CHAIN ID: A, *blue*) at binding site

Examining the bound chain structures in PDB database, it is observed that ligands do not always have a stable binding with protein chains at binding site, such as PDB ID: 2j22, CHAIN ID: A (Fig. 5). For this kind of bound structures, binding sites could not be computed directly based on their ligands. Thus, Stability of Complex is introduced into our algorithm to avoid these situations.

**Fig. 6:**
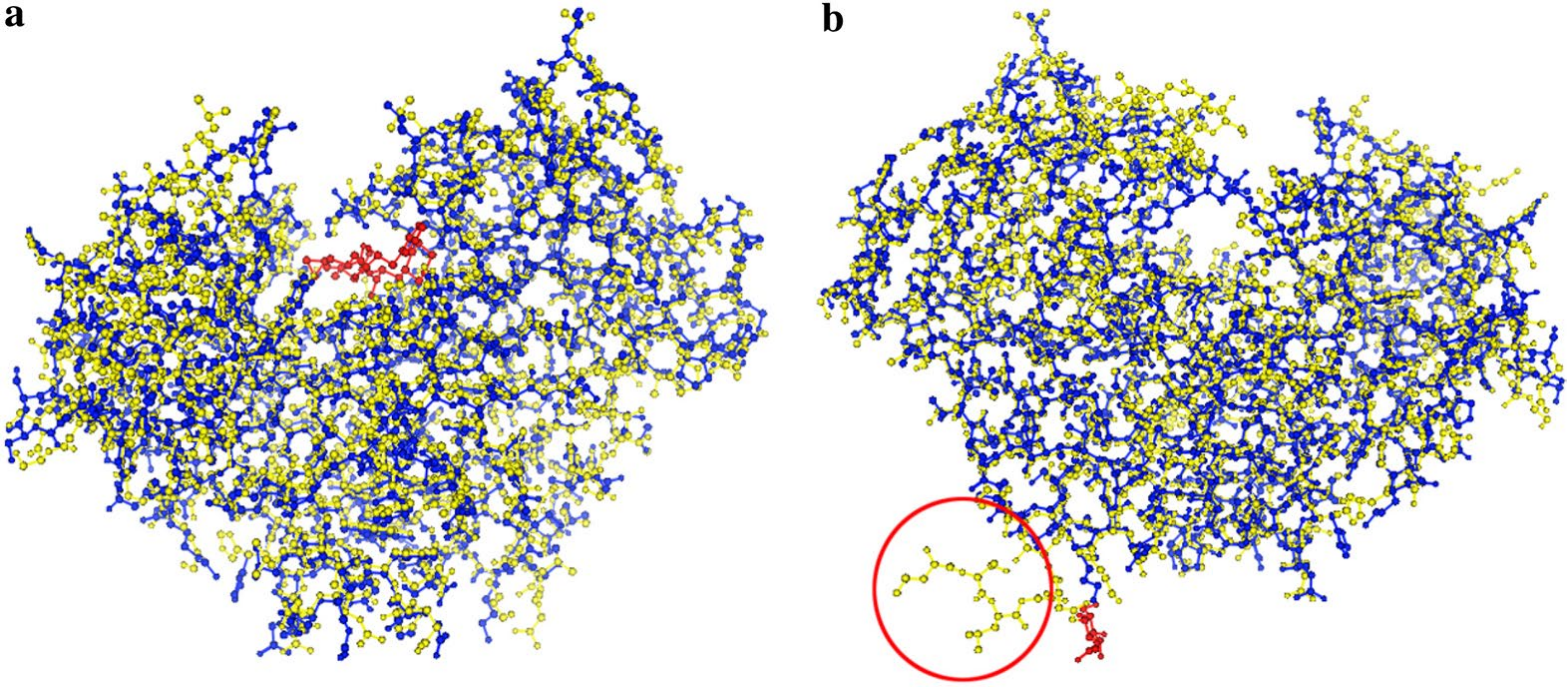
**a** Unbound chain (PDB ID: 1bbs, CHAIN ID: A, *blue*) and related appropriate template (PDB ID: 1hrn, CHAIN ID: B, *yellow*). After mapping the ligand (03D, *red*) in template to unbound chain, a new stable bound structure is formed with the tightly binding between the ligand and unbound chain. The top 20 templates at most ranked according to the similarity would be subsequently clustered. **b** Unbound chain (PDB ID: 1bbs, CHAIN ID: A, *blue*) and related appropriate template (PDB ID: 3g6z, CHAIN ID: A, *yellow*). After mapping the ligand (NAG, *red*) in template to unbound chain, a new stable bound structure could not be formed. The reason is that there are more residues (see the *red circle*) in template than unbound chain which have a close connection with the ligand

Looking for similar templates by structural alignments is needed for unbound chains which have no ligands to compute the binding site. In the process of structural alignment and ligand mapping successively, ligand in template may not have a stable bind with unbound chain (Fig. 6a, b). Likewise, Stability of Complex is employed here to decide whether ligand from template and unbound chain can form a new stable bound structure.

Similarly, Stability of Complex is introduced to build a template database (see details in Fig. 2), which reduced the number of bound structures from 117,823 to 101,315 with 14 % structures removed. Not only improved the quality of template database, this operation also reduced the number of time-consuming structural alignments.

### An Optimized Multiple-Templates Clustering method

Similar to FINDSITE [31], 3DLigandSite [25] and COFACTER [19], the prediction accuracy of our algorithm is improved by Optimized Multiple-Templates Clustering. However, in other works, the cluster number is required in previous algorithms, which actually could not be obtained before computing. In addition, the distances between ligands in each cluster have no reasonable physical meaning. In our algorithm, this deficiency is overcome by defining a new constraint, which restrict that the distances between geometric centers of all the ligands (for one binding site) in the same cluster should be less than a certain threshold (cluster radius). Ligands in multiple templates could be clustered automatically following the constraint with reasonable physical meaning, and there has no need to estimate cluster number before clustering.

Considering the space complexity of bound structure, cluster radius to be used is optimized based on test set. For 48 unbound dataset, threshold is set from 1.0 to 8.0 Å to compute the accuracy of the Top1 and Top3. Table 6 shows the accuracy computed with different cluster radius, and the accuracies of the Top1 range from 72.3 to 85.4 %. It’s worth noting that the accuracy of our algorithm with any cluster radius is higher than that of other algorithms (Tables 2, 6).

**Table 6:** Comparison of prediction accuracies using Optimized Multiple-Templates Clustering with different cluster radius with 48 unbound dataset

Threshold (Å)	Top1	Top3	Threshold (Å)	Top1	Top3
1	0.792	0.958	5	0.854	0.938
2	0.837	0.958	6	0.792	0.918
*3*	*0.854*	*0.958*	7	0.723	0.867
4	0.853	0.938	8	0.754	0.876

Result in Table 6 indicates that the Top1 and Top3 have highest prediction accuracies with 48 unbound dataset, when cluster radius is set to 3.0 Å. Thus, 3.0 Å is set as the default parameter by *bSiteFinder* in Optimized Multiple-Templates Clustering.

## Conclusions

*bSiteFinder* as a protein-binding sites prediction server was developed based on the largest database of bound templates so far with stringent quality control. Each protein chain submitted would be processed by following steps: (1) Binding sites prediction of high quality bound protein; (2) Binding sites prediction of unbound protein with bound templates of same Homology Indexing; (3) Binding sites prediction of unbound protein with bound templates of Chain Length Indexing. Any protein chain submitted could receive the results of binding sites via efficient computation. By introducing Homology Indexing, Chain Length Indexing, Stability of Complex and Optimized Multiple-Templates Clustering into our algorithm, the efficiency of our server have been significantly improved. What’s more, the accuracy was approximately 2–10 % higher than that of other algorithms for the test with either bound dataset or unbound dataset. For 210 bound dataset, *bSiteFinder* achieved high accuracies up to 94.8 % (MCC 0.95). For another 48 bound/unbound dataset, *bSiteFinder* achieved high accuracies up to 93.8 % for bound proteins (MCC 0.95) and 85.4 % for unbound proteins (MCC 0.72). An online *bSiteFinder* server is freely available at http://binfo.shmtu.edu.cn/bsitefinder/, and the source code is provided at the methods page. Our work lays a foundation for functional annotation of protein and structure-based drug design. With ever increasing numbers of three-dimensional protein–ligand complex structures, our server should be more accurate and less time-consuming.

## References

[1] Greer J, Erickson JW, Baldwin JJ, Varney MD (1994). Application of the three-dimensional structures of protein target molecules in structure-based drug design. J Med Chem.

[2] Fuller JC, Burgoyne NJ, Jackson RM (2009). Predicting druggable binding sites at the protein-protein interface. Drug Discov Today.

[3] Mandal S, Moudgil M, Mandal SK (2009). Rational drug design. Eur J Pharmacol.

[4] Rausell A, Juan D, Pazos F, Valencia A (2010). Protein interactions and ligand binding: from protein subfamilies to functional specificity. Proc Natl Acad Sci USA.

[5] Laurie ATR, Jackson RM (2006). Methods for the prediction of protein-ligand binding sites for structure-based drug design and virtual ligand screening. Curr Protein Pept Sci.

[6] Honma T (2003). Recent advances in De novo design strategy for practical lead identification. Med Res Rev.

[7] Pradeep H, Rajanikant GK (2014). Computational prediction of a putative binding site on Drp 1: implications for antiparkinsonian therapy. J Chem Inf Model.

[8] Xiao X, Min JL, Lin WZ, Liu Z, Cheng X, Chou KC (2015). iDrug-Target: predicting the interactions between drug compounds and target proteins in cellular networking via benchmark dataset optimization approach. J Biomol Struct Dyn.

[9] Levitt DG, Banaszak LJ (1992). POCKET: a computer graphies method for identifying and displaying protein cavities and their surrounding amino acids. J Mol Graph.

[10] Hendlich M, Rippmann F, Barnickel G (1997). LIGSITE: automatic and efficient detection of potential small molecule-binding sites in proteins. J Mol Graph Model.

[11] Brady GP, Stouten PFW (2000). Fast prediction and visualization of protein binding pockets with PASS. J Comput Aid Mol Des.

[12] Laskowski RA (1995). Surfnet—a program for visualizing molecular-surfaces, cavities, and intermolecular interactions. J Mol Graph.

[13] Weisel M, Proschak E, Schneider G (2007). PocketPicker: analysis of ligand binding-sites with shape descriptors. Chem Cent J.

[14] Dai TL, Liu Q, Gao J, Cao ZW, Zhu RX (2011). A new protein-ligand binding sites prediction method based on the integration of protein sequence conservation information. BMC Bioinform.

[15] Laurie ATR, Jackson RM (2005). Q-SiteFinder: an energy-based method for the prediction of protein-ligand binding sites. Bioinformatics.

[16] Ngan CH, Hall DR, Zerbe B, Grove LE, Kozakov D, Vajda S (2012). FTSite: high accuracy detection of ligand binding sites on unbound protein structures. Bioinformatics.

[17] Lichtarge O, Bourne HR, Cohen FE (1996). An evolutionary trace method defines binding surfaces common to protein families. J Mol Biol.

[18] Brylinski M, Skolnick J (2008). A threading-based method (FINDSITE) for ligand-binding site prediction and functional annotation. Proc Natl Acad Sci USA.

[19] Roy A, Yang JY, Zhang Y (2012). COFACTOR: an accurate comparative algorithm for structure-based protein function annotation. Nucleic Acids Res.

[20] Yang JY, Roy A, Zhang Y (2013). Protein-ligand binding site recognition using complementary binding-specific substructure comparison and sequence profile alignment. Bioinformatics.

[21] Liang SD, Zhang C, Liu S, Zhou YQ (2006). Protein binding site prediction using an empirical scoring function. Nucleic Acids Res.

[22] Sonavane S, Chakrabarti P (2010). Prediction of active site cleft using support vector machines. J Chem Inf Model.

[23] Xie ZR, Liu CK, Hsiao FC, Yao A, Hwang MJ (2013). LISE: a server using ligand-interacting and site-enriched protein triangles for prediction of ligand-binding sites. Nucleic Acids Res.

[24] Oh M, Joo K, Lee J (2009). Protein-binding site prediction based on three-dimensional protein modeling. Proteins.

[25] Wass MN, Kelley LA, Sternberg MJE (2010). 3DLigandSite: predicting ligand-binding sites using similar structures. Nucleic Acids Res.

[26] Ortiz AR, Strauss CEM, Olmea O (2002). MAMMOTH (Matching molecular models obtained from theory): an automated method for model comparison. Protein Sci.

[27] Fox NK, Brenner SE, Chandonia JM (2014). SCOPe: structural classification of proteins-extended, integrating SCOP and ASTRAL data and classification of new structures. Nucleic Acids Res.

[28] Prlić A, Yates A, Bliven SE, Rose PW, Jacobsen J, Troshin PV, Chapman M, Gao JJ, Koh CH, Foisy S (2012). BioJava: an open-source framework for bioinformatics in 2012. Bioinformatics.

[29] Huang BD, Schroeder M (2006). LIGSITEcsc: predicting ligand binding sites using the Connolly surface and degree of conservation. BMC Struct Biol.

[30] Matthews BW (1975). Comparison of the predicted and observed secondary structure of T4 phage lysozyme. *Biochimica et Biophysica Acta (BBA)*-*Protein*. Structure.

[31] Skolnick J, Brylinski M (2009). FINDSITE: a combined evolution/structure-based approach to protein function prediction. Brief Bioinform.

[32] Xie ZR, Hwang MJ (2012). Ligand-binding site prediction using ligand-interacting and binding site-enriched protein triangles. Bioinformatics.

